# The Moderating Role of Maternal Praise and Positivity in the Association Between Callous-Unemotional (CU) Traits and Later Aggression: A Prospective Study in Preschool Children in Colombia

**DOI:** 10.1007/s10578-022-01354-3

**Published:** 2022-06-16

**Authors:** Diana Obando, Jonathan Hill, Nicola Wright

**Affiliations:** 1 Department of Psychology, Universidad de La Sabana, Chia, Colombia; 2 Department of Psychology and Clinical Language Sciences, University of Reading, Reading, UK; 3 Faculty of Health, Psychology and Social Care, Manchester Metropolitan University, Manchester, UK

**Keywords:** Positive parenting, Callous-unemotional traits, Aggression, Conduct problems, Children, preschool

## Abstract

Our previous findings in the UK and Colombia show that CU traits predict later aggression specifically among children who are already aggressive. We hypothesised that this effect would be reduced in the presence of maternal praise and positivity. In a sample of 220 mothers and children from Colombia, mother–child interactions were coded for maternal praise and positivity, and mothers reported on children’s CU traits at age 3.5 and aggression at ages 3.5 and 5 years. The results show three-way interactions between CU traits, child aggression and observed parenting at age 3.5 years in the prediction of later child aggression, and two-way interactions indicating a protective effect of positive parenting in the high aggressive children. Based on our finding, it is plausible that positive parenting may modify the effect of CU traits in the highly vulnerable group of children who are already aggressive in early childhood.

**Supplementary Information:**

The online version contains supplementary material available at 10.1007/s10578-022-01354-3.

## Introduction

The construct of callous-unemotional (CU) traits has proved highly productive in the identification of important heterogeneity within conduct problems (CPs), and hence in understanding their aetiology, and maintenance, and ultimately in the identification of treatments [1]. CU traits may create vulnerability to CPs, and in particular aggression, by removing an internal source of restraint, empathy for others’ distress. In that case, the effect of CU traits on later aggression may be attenuated in the presence of alternative sources of restraint. These may be internal, for example, higher levels of physiological arousal [2] or increased mentalisation [3]. External restraint in the presence of elevated CU traits may also be provided by positive reinforcement for prosocial behaviours, or by warm supportive parenting which may promote the internalisation of social norms and prosocial behaviour [4, 5]. We have previously shown that CU traits at age 3.5 are associated with aggression at age 5.0 years specifically among children who are already aggressive [6]. In this paper we use observations of mother–child interactions at age 3.5 years and maternal report of child behaviours at ages 3.5 and 5.0 years and asked whether this association is attenuated by high maternal praise and high positivity.

### The Role of Positive Parenting in Relation to the Link Between CU Traits and Conduct Problems

A substantial literature, including a number of studies with genetically informed designs supports a role for positive parenting in the development of CU traits [7–9]. However, whilst there are theoretical accounts of the potential role of positive reinforcement and parental positivity in reducing CPs in children with CU traits [5, 10], the available evidence is inconsistent. Generally, prospective studies have failed to demonstrate that positive parenting moderates the association between CU traits and CPs. For example, studying a sample of aggressive children aged 9–12 years over 1 year, Pardini et al. [11] found that child reported parental warmth and involvement did not moderate associations between CU traits and later antisocial behaviour. Another study by Hyde et al. [12] which used a broad measure of observed positive parenting that included contingent use of praise with a general population high-risk sample of 364 children at age 2–4 years also found no evidence for moderation. By contrast, moderation was observed prospectively in a lower risk sample of 100 two parent families in which positive parenting was observed over multiple contexts at ages 38 and 52 months, CU traits and externalising behaviours were assessed at 67 months and externalising behaviours again at 80 and 100 months. Kochanska et al. [5] found that positive parenting reduced the prospective association between CU traits and later externalising behaviours. The positive parenting coded in this study was more dyadic than in other studies, reflecting shared positive affect and shared responsiveness between parent and child. However, this study collected both mother and father observed parenting and mother and father report of externalising and only two out of four interactions were significant.

In one study cross-sectional moderation was found that was not evident at follow up. Kroneman et al. [13] found that low maternal warmth predicted decreasing levels of CPs among girls with high CU behaviour (N = 1233; aged 7–8 years old at baseline) but the interaction was no longer significant after 5 years. Moderation has also been shown in several solely cross-sectional studies. In a study by Pasalich et al. [14] of clinic referred boys aged 4–12 years, parental warmth assessed in the Five-Minute Speech Sample (FMSS) [15] was associated with lower CPs, specifically in the presence of elevated CU traits. Likewise in another cross-sectional study aged 4 to 12 years by Clark and Frick [16] using parent report in the Alabama Parenting Questionnaire (APQ) [17] positive reinforcement was associated with lower CPs specifically in the presence of high CU traits. Studies using latent profile analysis can also provide pointers to the role of positive parenting. In a study of 1366 children aged 7–11 years at recruitment using the APQ [18], positive reinforcement and positive involvement were examined separately using child and mother report on the APQ. Latent profile analysis using data from the three time points yielded five groups, including a high-CU traits only group (9.4%) and a high-CPs and CU traits group (7.2%). Mothers of children in the high-CU traits only group reported higher levels of involvement and positive reinforcement than those of the high-CPs and CU traits group, consistent with a protective role for positive parenting.

### CU traits in Low- and Middle- Income Countries

CU traits have almost exclusively been investigated in High-Income Countries (HICs) [19] and it is yet to be established whether the construct and proposed mechanisms generalize across cultures and socioeconomic conditions. Evidence from Low and Middle-Income Countries (LMICs) has mainly been provided from China. Support for the reliability and validity of the CU traits construct in Chinese school-age and adolescent children has been provided [20, 21]. In this sample of preschool Colombian children, we have reported on the reliability and factor structure of the main measure of CU traits in children, the Inventory of Callous-Unemotional traits [6] and provided evidence of validity in relation to aggression. A cross-country comparison study of nine different HIC and LMIC societies (China, Colombia, Italy, Jordan, Kenya, the Philippines, Sweden, Thailand, or the United States) [22] indicated that Colombian parents reported higher levels of warm parenting than the grand mean across countries, but there were no significant differences in the association between warmth and child aggression. In unpublished data from the sample analysed here we have shown associations between parent-report of positive parenting and child CU traits, and further evidence for specificity in associations between positive parenting and CU traits and negative parenting and child ODD symptoms [2, 23]. Therefore, whilst there is evidence for a difference in levels of parenting the existing limited evidence supports similar associations between positive parenting, CU traits and aggression in LMIC settings. However, no study in a LMIC has examined whether positive parenting moderates the association between CU traits and CPs. In a HIC outside of a Western setting, and with adolescents, Sng et al. [24] examined moderation by negative parenting practices only and found evidence for moderation.

In sum, the existing evidence on whether positive parenting moderates the association between CU traits and aggression is inconsistent, with more support found for moderation in cross-sectional studies and limited evidence from prospective studies. Recent findings from the sample analysed here, and from the UK Wirral Child Health and Development Study have indicated that one possible explanation is that in young children the effect of CU traits is mainly seen among those who are already aggressive [6]. In that case we need to examine the protective effect of positive parenting, specifically in this group. In the present study we therefore examined whether there is an effect of positive parenting in attenuating the association between CU traits and later aggression, specifically among children who are already aggressive. To test this, we modelled the three-way interaction between maternal positive parenting, CU traits and child aggression at age 3.5 years in the prediction of aggression at age 5 years. Positive parenting was assessed as observed maternal praise for following instructions, and warmth and reciprocity during play.

## Methods

### Participants and Procedure

The *La Sabana Parent–Child Study* participants were recruited through Facebook groups likely to be used by young mothers, such as ‘Latin Women League’ and ‘More Moms Colombia’. It is estimated that 91% of those between the ages of 14 and 65 in Colombia access Facebook [25]. Parents who responded to the online study information (n = 344) were contacted to discuss participation. Of these, 40 were excluded as the children did not meet the age inclusion criteria of age 3–4 years. Of the remaining 304, 235 (77.3%) provided informed consent to take part in the study and full data. For the baseline assessment, 235 families with children of 3.5 years (M = 3.31, SD = 0.48) participated, 48% girls/52% boys, and the mothers’ average age was 30.04 years (SD = 6.29) which is similar to general population statistics, where the average age at first pregnancy is 28 years old [26]. At follow-up, 18 months later, 220 (93%) participants provided data for the analyses presented here (mean age = 4.86; SD = 0.42; 51% girls/49% boys). The study was approved by the Research and Ethical Committee of the Psychology Department at La Sabana University.

Participants were recruited from three Colombian regions, each with different cultural and demographic features. The Pacific (n = 69) and Caribbean (n = 70) regions are characterized by high levels of poverty and extensive numbers of Afro-Colombian and Indigenous inhabitants, while the Central region (n = 96) has the lowest levels of poverty in the country, and it is predominantly mestizo (mix of European and Indigenous) [27]. Overall, 15% of the participants lived in rural areas which is similar to national statistics [28]. The majority (77%) were two parent families and 45% of the sample belonged to the lowest two household income classifications, based on the Colombian government system that classifies households into six categories (1 the lowest) determined by housing conditions and basic public services, such as sewerage and water supply [29]. Classifications 1 and 2 receive subsidies from the Colombian government. The rate of low socioeconomic status (SES) was similar to national statistics [28]. Regarding participants ethnicity, 38% mothers identified as mestizo, 9% Afro-Colombian, 5% Indigenous, 13% from ‘other’ ethnic groups, and 35% did not identify themselves as belonging to a specific ethnic group. The prevalence of self-reported Indigenous or Afro-Colombian status is close to the national estimate [30]. The sample differed from national estimates on the proportion with a university degree, with 50% of mothers and 48% of fathers with university degrees, compared to 22% in the general population [31].

Participants were recruited across three contrasting regions, and a widely available medium was used for recruitment, yielding a sample that in many aspects was representative of the general population of Colombia. The rate of low socioeconomic status (SES; 45%) was similar to those in published studies in Colombia [28], and the numbers from rural districts (15%) were similar to national statistics (15) [28]. The prevalence of self-reported Indigenous or Afro-Colombian status (14%) was very close to the national estimate of 14.4% [30]. A third of the sample reported experiencing community violence or displacement. The sample differed, however, in that 50% of mothers and 48% of fathers had a university degree, contrasted with 22% in the general population [31]. Thus, although we contacted parents through social media almost universally accessed by the general population, participation was probably biased towards more highly educated parents, limiting the generalizability of the findings.

The sample analysed here represents participants who provided questionnaire data at age 3.5 and 5.0 years and mother–child observational data at 3.5 years (N = 220).

### Measures

#### Parental Report of Callous-Unemotional Traits

CU traits were measured at 3.5 years and at 5.0 years using the Spanish version of the parent-report preschool Inventory of Callous-Unemotional Traits (ICU) [32]. This inventory has 24 items scored using a 4-point scale (0 = not at all true, 1 = somewhat true, 3 = very true, and 4 = definitely true). Evidence for the reliability and validity of the ICU total score in preschool children in a HIC setting has been provided from a cross-sectional study by Kimonis et al. [33]. In a previous publication from this Colombian sample, we have replicated the best-fitting factor structure reported in Kimonis, a 12-item two-correlated factor structure, and found similar internal reliability and provided evidence for validity in relation to aggression [6]. Similar to Willoughby et al. [34] (2015) we have not been able to replicate the bi-factor structure of the ICU. However, in line with the recommendations of Ray et al. [35] and to retain comparability to the majority of the literature, we conducted the main analysis using the total 24-item ICU scale but report the results using the 12-item total in the appendix. The internal consistency in this sample for the 12-item total is α = 0.71 at age 3.5, and α = 0.78 at age 5 years, and for the 24-item total it is α = 0.82 at age 3 years, and α = 0.85 at age 5 years. The Spanish version of the inventory was shared by the authors, who approved its use in the present study.

#### Parental Report of Aggressive Behaviour: Child Behaviour Checklist (CBCL) [36]

The Spanish version of the CBCL for children aged 1.5 to 5 years was used to assess aggressive behaviours. In our data the aggressive behaviour syndrome scale had an internal consistency of 0.85 for the baseline and 0.88 for the follow-up, values that are like those previously reported with Colombian children (α = 0.86) [37].

#### Observation of Mother–Child Interactions

Video recordings were made of mothers and children in a standardized procedure used previously in National Institute for Child Health and Development studies (NICHD) [38]. Parents and children were provided with three bags of toys which they are asked to play with in a pre-set order over 15 min, with a pre-agreed signal the child is asked to tidy up the toys. Mother–child play was rated using the Parent–Child Interaction System (PARCHISY) [39] generating a parental positivity score as the average of *positive affect* (e.g., smiling and laughter: warm, friendly voice, inquisitive voice, looking at what the child is doing) and *reciprocity* (e.g., shared positive affect, eye contact and turn taking interaction) scales [40]. Unlabelled praise during tidy-up was assessed using the Dyadic Parent–Child Interaction Coding System (DPICS) [41] by identifying contingent neutral comment such as “that’s it”, “ok” or “keep going”, and contingent expressions of gratitude such as “thank you” (see appendix Table A1). Research assistants in Colombia were trained by reliable UK researchers, and high agreement between them on 30 independently rated recordings in English from the UK was achieved (all Intraclass Correlation Coefficients [ICCs] ≥ 0.78). There were too few instances of labelled praise in either UK or Colombian studies for reliability analyses.

**Table 1:** Summary of multiple linear regression models predicting age 5.0 aggression from maternal praise, CU traits and aggression

	ΔR^2^	p	Variable	β	p
Block 1	0.15	< 0.001	Child sex	0.11	0.082
			Higher family income	− 0.03	0.663
			Married/cohab parents	− 0.05	0.483
			Pacific region	− 0.01	0.915
			Caribbean region	− 0.01	0.864
			Maternal mood age 5.0	0.36	< 0.001
Block 2	0.18	< 0.001	Aggression age 3.5	0.39	< 0.001
			CU traits age 3.5 years	0.19	0.005
			Maternal praise	0.11	0.080
Block 3	0.01	0.617	Aggression × CU traits 3.5	0.06	0.296
			Aggression × praise 3.5	− 0.01	0.884
			CU traits × praise	− 0.04	0.663
Block 4	0.01	0.047	Aggression × praise × CU traits	− 0.18	0.047

Block 1 effects were generated from a model with only Block 1, Block 2 effect from a model with Block1 plus Block 2, and Block 3 effect from a model with all three Blocks

#### Covariates

The Spanish version of the Edinburgh Postnatal Depression Scale (EPDS) [42] was used to assess maternal mood at age 5 to account for possible mood related biases in reporting. The EPDS has 10 items scored on a 4-point scale. This measure is widely used during the prenatal and postnatal periods with parents of young children [43]. Internal consistency of the EPDS was good both in a previous study of Colombian women (α = 0.78) [44] and in this study (α = 0.83). Child sex, household income (operationalised as a binary variable, 0 = lowest two DANE family income classifications, 1 = all other income classifications), single- versus two-parent family structure (0 = single parent, 1 = two cohabiting parents) and dummy variables for Caribbean and Pacific regions were included as covariates in regression analyses.

### Data Analyses

Stata version 16 was used for the statistical analyses. Following square root transformations for skewed variables, bivariate associations between the study variables were examined using Pearson, point-biserial and tetrachoric correlations. Maternal praise was highly skewed and therefore a binary variable reflecting 0 = no praise, and 1 = 1 or more instances of praise was used for analysis. Maternal praise and positivity were examined in two separate regression models. The models were estimated in three blocks. Block 1 included demographic covariates and parental mood at age 5 years, Block 2 included age 3.5 years child aggression, age 3.5 years CU traits, and parenting, Block 3 included the two-way interactions terms, and Block 4 the three-way interaction between age 3.5 years aggression, CU traits and parenting, testing at each stage whether the addition of a block significantly increased the explained variance. The three-way interactions were then explored by examining the two-way interactions between CU traits and parenting at high and low levels of age 3.5 aggression, indexed using a median split.

## Results

Bivariate associations between the transformed study variables and descriptive statistics for untransformed variables are presented in the Online Appendix Table A2.

Table 1 shows separate models for maternal report of aggression regressed on to Block 1 variables, then Block 2 after accounting for Block 1, Block 3 accounting for Block 1 and 2 and similarly Block 4 after accounting for Block 1, 2 and 3 variables, so that all the reported coefficients are directly interpretable. After accounting for sociodemographic confounders and maternal mood at time of reporting outcome in Block 2, age 3.5 years CU traits, aggression and, unexpectedly, maternal praise, all significantly predicted increased age 5.0 aggression. In Block 3, none of the two-way interaction terms was significant. However, in Block 4, the three-way interaction between CU traits, aggression and praise was significant (p = 0.025). In Table 2, we show the two-way interaction between CU traits and praise in groups below and above the median on age 3.5 years aggression.

**Table 2:** Summary of multiple linear regression models predicting age 5.0 years aggression from age 3.5 years CU traits and maternal praise in high and low aggression groups

Variable	Low Aggression				High Aggression			
	ΔR^2^	p	β	p	ΔR^2^	p	β	p
Block 2	0.05	0.075			0.09	0.004		
Maternal praise			0.14	0.150			− 0.02	0.831
CU traits			0.20	0.046			0.33	< 0.001
Block 3	0.02	0.121			0.02	0.143		
CU traits × maternal praise			0.21	0.121			− 0.18	0.143

Block 1 effects were generated from a model with only Block 1, Block 2 effect from a model with Block1 plus Block 2

**Fig. 1:**
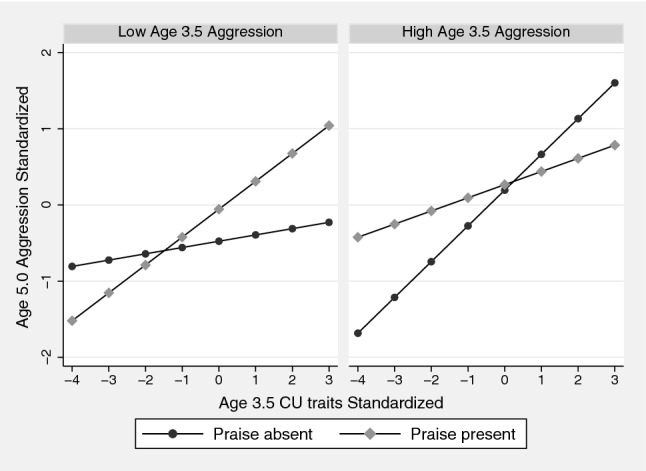
The association between age 3.5 CU traits and age 5.0 aggression at high and low praise in children above and below the median on age 3.5 years aggression

Whilst the two-way interaction in the high aggression group was not conventionally significant, it can be seen in the right panel of Fig. 1 that the association between age 3.5 years CU traits and age 5.0 aggression was attenuated in the children with high praise. Unexpectedly, whilst the two-way interaction in the low aggression group was also non-significant, it can be seen in Fig. 1 that the association between age 3.5 CU traits and later aggression was stronger in children whose mothers had praised them more. When we repeated this analysis using the short 12-item ICU (shown in Online Appendix Table A3), the three-way interaction was no longer significant (p = 0.056), which arose because this counterintuitive interaction was substantially attenuated (reduced from β = 0.22, p = 0.112, to β = 0.07, p = 0.610). The size and significance of the hypothesised two-way interaction in the high aggression group was slightly increased (− 0.24, p = 0.061).

We then estimated the same regression models to test for moderation by maternal positivity, shown in Table 3. There was a non-significant (p = 0.166) three-way interaction between CU traits, aggression and maternal positivity, which arose from different effects of positivity in children showing low vs high aggression at age 3.5 years. Among those with low aggression, maternal positivity did not modify the association between CU traits and later child aggression (p = 0.884), while there was a moderator effect in the high aggression group (p = 0.040; Table 4).

**Table 3:** Summary of multiple linear regression models predicting age 5.0 years aggression from maternal positivity, CU traits and aggression

	ΔR^2^	p	Variable	β	p
Block 1	0.15	< 0.001	Child sex	0.11	0.082
			Higher family income	− 0.04	0.663
			Married/cohab parents	− 0.05	0.462
			Pacific region	− 0.01	0.915
			Caribbean region	− 0.01	0.864
			Maternal mood age 5.0	0.36	< 0.001
Block 2	0.18	< 0.001	Aggression age 3.5	0.36	< 0.001
			CU traits age 3.5 years	0.18	0.008
			Maternal positivity	− 0.06	0.302
Block 3	0.02	0.140	Aggression × CU traits 3.5	0.08	0.218
			Aggression × positivity 3.5	0.12	0.066
			CU traits × positivity	− 0.09	0.183
Block 4	0.01	0.166	Aggression × positivity × CU traits	− 0.10	0.166

Block 1 effects were generated from a model with only Block 1, Block 2 effect from a model with Block1 plus Block 2, and Block 3 effect from a model with all three Blocks

**Table 4:** Summary of multiple linear regression models predicting age 5.0 years aggression from age 3.5 years CU trait and maternal positivity in the high and low aggression groups

	Low Aggression				High Aggression			
Variable	ΔR^2^	p	β	p	ΔR^2^	p	β	p
Block 2	0.05	0.073			0.09	0.003		
Maternal positivity			− 0.14	0.187			− 0.02	0.883
CU traits			0.19	0.062			0.33	0.001
Block 3	0.01	0.844			0.03	0.040		
CU traits × maternal positivity			0.02	0.884			− 0.20	0.040

Block 1 effects were generated from a model with only Block 1, Block 2 effect from a model with Block1 plus Block 2

**Fig. 2:**
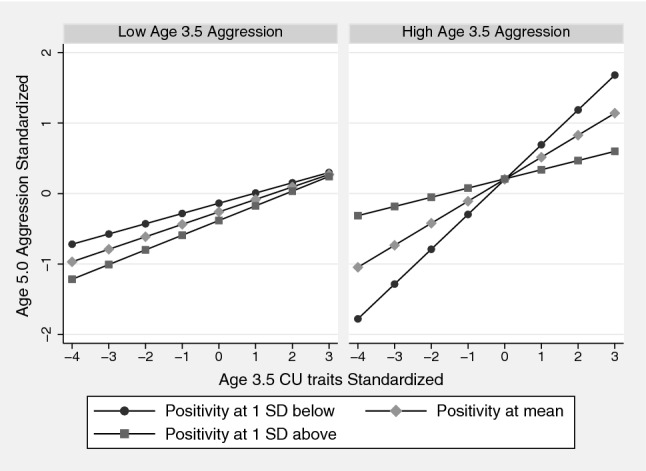
The association between age 3.5 CU traits and age 5.0 aggression at three levels of maternal positivity (at mean and 1 SD below and above) in children above and below the median on age 3.5 years aggression

Figure 2 shows the association between age 3.5 CU traits and age 5.0 aggression at three levels of age 3.5 maternal positivity (at mean and 1 SD above and below). It can be seen that in children with high aggression at age 3.5 years, the association between CU traits and age 5.0 aggression was attenuated in those whose mothers had shown high positivity. The analysis was repeated using the 12-item ICU (reported in the Online Appendix Tables A5 and A6) and the pattern of the results were the same, but the size of the effects was reduced, and all were non-significant.

## Discussion

Against a background of inconsistent evidence as to whether positive parenting moderates the association between CU traits and CPs, we asked whether the protective effect of positive parenting may be seen specifically in children who are already aggressive. In relation both to maternal praise and maternal positivity (warmth and reciprocity), in children who were already aggressive, the prospective association between CU traits at age 3.5 years and aggression at 5 years was reduced in the presence of positive parenting, consistent with our hypotheses.

This was the first study to examine whether moderation by positive parenting of the association between CU and aggression is confined to children already aggressive [5, 11, 12]. Our results provide evidence for the hypothesis that both maternal praise and maternal positivity moderate the association between CU traits and aggression, but only in children who are already aggressive. Among children already aggressive at age 3.5 years, standardised betas for the two-way interactions between both parenting variables (praise and positivity) and CU traits predicting age 5.0 years aggression were very similar, reflecting that CU traits predicted later aggression only in the presence of low positive parenting. We interpret the values of ‘p’ for the interactions, which fell either side of the conventional significance level, mainly in the light of the very similar effect sizes for the interactions, consistent with American Statistical Society advice [45].

Although there was a significant three-way interaction for maternal praise in analyses using the 24-item measure of CU traits (ICU), this arose in part because in the children with low aggression at age 3.5 years CU traits were more strongly associated with later aggression in the presence of high maternal praise. This puzzling pattern of associations did not emerge when the analyses were conducted using the 12-item ICU, and the corresponding 3-way interaction, while still evident also became non-significant. Therefore, our findings provide good evidence that positive parenting attenuates the association between CU traits and later aggression, in children who are already aggressive, but they do not provide clear evidence for a contrast with children with lower levels of aggression.

It is possible that the differences in the findings when using the 12-item and 24-item versions of the ICU are explained by items omitted from the shorter version which may not best reflect the CU traits construct. The 12-item total has been shown to be the best fitting factor structure for the ICU in pre- and school-age children. It removes five of the six items from the unemotional scale, which have been found to show opposite to expected associations with external correlates and has been speculated to assess shy and withdrawn behaviour rather than unemotionality as it is conceptualised in CU traits [46]. The other items excluded from the 12-item version assess unconcern about performance. Whilst this component of CU traits has been included in the DSM *limited prosocial specifier* CPs, it is not included in most conceptualisations of CU traits or psychopathic traits [47–49]. It may be that the unconcern about performance items are less relevant to pre- and school-aged children. A a similar 11-item version (excluding the one remaining unemotional item) has been shown to be the best fitting factor structure of the ICU in Chinese adolescents and undergraduate students [20, 21] and Chinese and UK 10–11 years old [50]. These results suggest that the excluded items are less central or not part of the CU traits construct.

As we noted earlier, there are strong theoretical reasons to suggest that positive parenting will be protective for CPs or aggression in children with elevated CU traits. Drawing on behavioural and neurological evidence that individuals with CU traits are less responsive to punishment but may be more responsive to reward [51, 52]. Reidy et al. [10] have argued specifically for a role for positive reinforcement of prosocial behaviour, suggesting that it may be a particularly relevant restraint on antisocial behaviour amongst individuals with CU traits due to their reward-dominant processing style. Work on the development of conscience [4, 53, 54] has suggested that warm and mutually responsive positive parenting may reduce antisocial behaviour by promoting the internalisation of social rules, but only in children who are temperamentally fearless and thus may be at risk of aggressive behaviour. Whilst some existing studies have examined the moderation between positive parenting and CU traits in CP on aggressive samples [11], the majority have used general population or samples at elevated risk due to sociodemographic variables and have been based on the expectation that CU traits are related to later aggression as a main effect. Our previous findings across Colombian and UK samples were consistent with that assumption but also indicated that the strength of the association between CU traits and later aggression was much stronger in the presence of pre-existing aggression. This finding that we replicated across informants and in Colombian and UK samples created a new context for the study of the role of positive parenting. This heterogeneity may account at least in part for the negative findings in relation to CU traits as a main effect. Our previous findings suggest that among children with low levels of aggression, CU traits do not present an increased risk for aggression, and therefore there is little or no effect to be modified by quality of parenting. The findings reported here are consistent with, but do not provide conclusive evidence in support of, this explanation.

The strengths of the study include the use of a prospective design in which parenting at 3.5 years was observed, thus reducing possible shared method variance effects on the rating of parenting and psychopathology. Further, this is the first study to examine whether positive parenting moderates the association between CU traits and CPs in a LMIC setting. Both positivity and praise showed the expected negative association with CU traits found in HICs. The participants were recruited across three contrasting regions, and a widely available medium was used for recruitment, yielding a sample that in many aspects was representative of the general population of Colombia. The rate of low socioeconomic status was similar to those in published studies in Colombia [28], and the numbers from rural districts were similar to national statistics (15) [28]. The prevalence of self-reported Indigenous or Afro-Colombian status was very close to the national estimate [30].

A limitation of the study is that 50% of mothers and 48% of fathers had a university degree, contrasted with 22% in the general population of Colombia [31]. Thus, although we contacted parents through social media almost universally accessed by the general population, participation was probably biased towards more highly educated parents, limiting the generalizability of the findings. A further limitation is that it is possible that associations between parenting and child aggression arose because mothers who show less positive parenting perceive their children more negatively. The study findings would have been strengthened by collecting additional informants on child behaviour. Additional limitations include that statistical power for the hypothesised three-way interaction was modest, which created challenges in evaluating statistical significance, and created risks not only of Type 1 but also Type 2 errors.

Future directions from this research include examination of whether the moderation of CU traits by positive parenting in aggressive children persists over longer follow up periods. Evidence has shown that the association between parenting and both CPs and CU traits is bidirectional [8] and this may change over development with certain parenting behaviours potentially becoming more or less relevant to the emergence versus the maintenance of CU traits and CPs [46]. Analyses which can take into account parenting behaviours at the outcome age, and continuity or discontinuity in parenting behaviour over time, are needed. Finally, recent work has indicated that psychopathic traits—the constellation of CU traits, grandiose traits and sensation seeking—are a better predictor of future antisocial behaviour than CU traits alone [47]. The majority of child literature has focused on CU traits alone, and more work needs to be done to examine whether the same mechanisms underpin psychopathic traits. Future studies should examine first whether psychopathic traits create vulnerability is already aggressive children and if they do whether this is moderated by positive parenting.

The present findings indicate that further study of the role of parenting in children who have high levels both of CU traits and aggression, is warranted. On the one hand this seems to be a particularly high-risk group, and on the other, they might prove to be particularly responsive to positive parenting, and hence perhaps to interventions to promote positive parenting. In a recent publication, Kimonis et al. [55] reported that an adapted parent–child interaction therapy designed to promote positive parenting produced reductions in CP in pre-schoolers with CU traits, which were sustained at a 3-month follow-up. Future investigations which employ longer term follow-up are needed to inform whether targeting positive parenting will result in persistent reductions in CPs in children with CU traits.

## Summary

The evidence as to whether positive parenting attenuates the association between CU traits and conduct problems is inconsistent. Building on our previous finding that CU traits predict later aggression specifically in children who are already aggressive, we asked whether positive parenting attenuates this association between CU traits and later aggression. The sample comprised 220 mothers of children (48% female) recruited at age 3.5 years through social media across three regions in Colombia. Mother–child interactions were coded for maternal positive reinforcement and positivity, and mothers reported on their children’s CU traits and aggression at age 3.5 years, and aggression again at 5.0 years. In multiple linear regression, there was a significant three-way interaction between maternal praise, CU traits and aggression (p = 0.025). In the presence of high aggression, high maternal praise was associated with a reduced association between CU traits and later aggression, although this two-way interaction was not significant (p = 0.097). The three-way interaction between maternal positivity, CU traits and aggression was not significant (p = 0.093), but similar to maternal praise, maternal positivity had a protective effect among children already aggressive, and the two-way interaction was significant (p = 0.045). The findings provide evidence that positive parenting, both praise and maternal positivity, moderates the association between CU traits and later aggression in children who are already aggressive. This topic has great potential clinical relevance and future longitudinal studies with an intervention design are required.

### Supplementary Information

Below is the link to the electronic supplementary material.

Supplementary file1 (DOCX 27 KB)
