# Supplementary material for: The Moderating Role of Maternal Praise and Positivity in the Association Between Callous-Unemotional (CU) Traits and Later Aggression: A Prospective Study in Preschool Children in Colombia
Source: Child Psychiatry Hum Dev. 2022 Jun 16;55(1):14–23. doi: 10.1007/s10578-022-01354-3 (PMC10796412; doi:10.1007/s10578-022-01354-3)
Supplement: Supplementary file 1 — Supplementary file1 (DOCX 27 KB) [file 10578_2022_1354_MOESM1_ESM.docx]

**Appendix**

**Table A1** Distribution of unlabelled praise instances observed during parent-child interactions.

| Instances | Frequency | Percentage |
| --- | --- | --- |
| 0 | 112 | 47.7 |
| 1 | 52 | 22.1 |
| 2 | 27 | 11.5 |
| 3 | 11 | 4.7 |
| 4 | 15 | 6.4 |
| 5 | 9 | 3.8 |
| 6 | 4 | 1.7 |
| 7 | 1 | .4 |
| 8 | 2 | .9 |
| 9 | 1 | .4 |
| 12 | 1 | .4 |
| Total | 235 | 100.0 |

**Table A2** Bivariate associations, Pearson’s and tetrachoric, between transformed study variables and descriptive statistics on untransformed variables

|  | Positivity | High praise | Aggr. 3.5 yrs. | Aggr. 5.0 | ICU-24 age 3.5 | ICU-12 age 3.5 | Maternal mood | High-Income | Two- parent | Male sex |
| --- | --- | --- | --- | --- | --- | --- | --- | --- | --- | --- |
| Praise | .12^+^ |  |  |  |  |  |  |  |  |  |
| Aggression 3.5 | -.15 | -.16* |  |  |  |  |  |  |  |  |
| Aggression 5.0 | -.16* | -.02 | .49** |  |  |  |  |  |  |  |
| ICU-24 3.5 yrs. | -.18* | -.14* | .40** | .40** |  |  |  |  |  |  |
| ICU-12 3.5 years | -.13* | -.16* | .38** | .36** | .91** |  |  |  |  |  |
| Maternal mood | -.04 | -.12^+^ | .39 | .35** | .28** | .24** |  |  |  |  |
| High-income | .01 | -.21* | -.13^+^ | -.12^+^ | -.21** | -.17** | -.23** |  |  |  |
| Two parents | -.02 | .12 | -.17* | -.10 | -.03 | -.02 | -.14 | .14 |  |  |
| Male sex | -.16* | .01 | .05 | .15* | .07 | .17** | -.03 | .01 | .05 |  |
| Mean | 3.27 |  | 1.36 | 16.01 | 13.85 | 7.11 | 6.17 |  |  |  |
| SD | 1.11 |  | .50 | 8.71 | 8.06 | 4.88 | 4.49 |  |  |  |
| N | 235 | 235 | 235 | 235 | 220 | 235 | 220 | 235 | 235 | 235 |
| % |  | 52% |  |  |  |  |  | 40% | 73% | 51% |

+ p <.08; * p <.05; ** p <.01; Aggr.= aggression; yrs.= years.

**Table A2** Summary of multiple linear regression models predicting age 5.0 aggression from age 3.5 years 12-item CU traits, aggression, and maternal praise

|  | ΔR^2^ | p | Variable | β | p |
| --- | --- | --- | --- | --- | --- |
| Block 1 | .15 | <.001 | Child sex | .11 | .082 |
|  |  |  | High income | -.03 | .663 |
|  |  |  | Married/Cohab parents | -.02 | .483 |
|  |  |  | Pacific region | -.10 | .915 |
|  |  |  | Caribbean region | -.06 | .864 |
|  |  |  | Maternal mood age 5.0 | .36 | <.001 |
| Block 2 | .18 | P<.001 | Aggression age 3.5 | .37 | <.001 |
|  |  |  | CU traits age 3.5 years | .28 | .002 |
|  |  |  | Maternal praise | .10 | .100 |
| Block 3 | .02 | .194 | Aggression X CU traits 3.5 | .17 | .032 |
|  |  |  | Aggression X praise 3.5 | .03 | .772 |
|  |  |  | CU traits X praise | -.13 | .122 |
| Block 4 | .01 | .082 | Aggression X praise X CU traits | -.15 | .082 |

*Note*: Block 1 effects were generated from a model with only Block 1, Block 2 effect from a model with Block1 plus Block 2, and Block 3 effect from a model with all three Blocks.

**Table A3** Summary of multiple linear regression models predicting age 5.0 aggression from age 3.5 years CU traits and maternal praise in high and low aggression groups

|  |  | Low Aggression | | | | High Aggression | | | |
| --- | --- | --- | --- | --- | --- | --- | --- | --- | --- |
| Variable | | ΔR^2^ | p | β | p | ΔR^2^ | p | β | p |
| Block 2 | | .04 | .122 |  |  | .10 | <.001 |  |  |
|  | Maternal praise |  |  | .14 | .156 |  |  | -.02 | .806 |
|  | CU traits |  |  | .17 | .096 |  |  | .34 | .001 |
| Block 3 | | .01 | .717 |  |  | .03 | .044 |  |  |
|  | CU traits X praise |  |  | .16 | .672 |  |  | -.26 | .041 |

*Note*: Block 1 effects were generated from a model with only Block 1, Block 2 effect from a model with Block1 plus Block 2.

**Table A4** Summary of multiple linear regression models predicting age 5.0 years aggression from age 3.5 years 12-item CU traits, aggression, and maternal positivity

|  | ΔR^2^ | p | Variable | β | p |
| --- | --- | --- | --- | --- | --- |
| Block 1 | .15 | <.001 | Child sex | .12 | .076 |
|  |  |  | Household income | -.02 | .817 |
|  |  |  | Family structure | -.05 | .462 |
|  |  |  | Pacific region | -.01 | .907 |
|  |  |  | Caribbean region | -.01 | .908 |
|  |  |  | Maternal mood age 5.0 | .37 | <.001 |
| Block 2 | .18 | P<.001 | Aggression age 3.5 | .36 | <.001 |
|  |  |  | CU traits age 3.5 years | .17 | .009 |
|  |  |  | Maternal positivity | -.07 | .262 |
| Block 3 | .02 | .173 | Aggression X CU traits 3.5 | .10 | .111 |
|  |  |  | Aggression X positivity 3.5 | .10 | .120 |
|  |  |  | CU traits X positivity | -.05 | .407 |
| Block 4 | .01 | .352 | Aggression X positivity X CU traits | -.06 | .352 |

*Note*: Block 1 effects were generated from a model with only Block 1, Block 2 effect from a model with Block1 plus Block 2, and Block 3 effect from a model with all three Blocks.

**Table A5** Summary of multiple linear regression models predicting age 5.0 years aggression from age 3.5 years CU traits and maternal positivity in high and low aggression groups

|  |  | Low aggression | | | | High aggression | | | |
| --- | --- | --- | --- | --- | --- | --- | --- | --- | --- |
| Variable | | ΔR^2^ | p | β | p | ΔR^2^ | p | β | p |
| Block 2 | | .04 | .126 |  |  | .10 | .002 |  |  |
|  | Maternal positivity |  |  | -.15 | .156 |  |  | -.04 | .663 |
|  | CU traits |  |  | .15 | .122 |  |  | .34 | .001 |
| Block 3 | | .01 | .799 |  |  | .01 | .189 |  |  |
|  | CU traits X maternal positivity |  |  | .03 | .799 |  |  | -.13 | .189 |

*Note*: Block 1 effects were generated from a model with only Block 1, Block 2 effect from a model with Block1 plus Block 2.
